# A contemporary review of the head-up tilt test: Utility and limitations

**DOI:** 10.1016/j.ihj.2025.03.014

**Published:** 2025-04-02

**Authors:** Ameya Udyavar, Jayaprakash Shenthar, Ajay Madhukar Naik, Dibbendhu Khanra, Vadivelu Ramalingam, Rahul Singhal, Dinesh Choudhary, Prabha Nini Gupta, B Hygriv Rao, Saurabh Mehrotra, Vanita Arora, Sanjeev Kathuria, Pawan Suri, David Benditt, Richard Sutton

**Affiliations:** a P.D. Hinduja National Hospital, Mahim, Mumbai, India; b Sri Jayadeva Institute of Cardiovascular Sciences and Research, Bengaluru, India; c Arrhythmia and Heart Failure Device Division, Marengo CIMS Hospital, India; d Queen Elizabeth Hospital, Birmingham, United Kingdom; e Department of Cardiology, Velammal Medical College Hospital, Madurai, India; f Cardiology and Cardiac Electrophysiology, Fortis Hospital, Jaipur, India; g Cardiology, S P Medical College, Bikaner, Rajasthan, India; h Sri Ramakrishna Mission Hospital Trivandrum, India; i Division of Pacing &Electrophysiology, KIMS Hospitals, Hyderabad, India; j Cardiology, PGIMER, Chandigarh, India; k Cardiac Electrophysiologist, Indraprastha Apollo Hospital, New Delhi, India; l GIPMER, New Delhi, India; m Global Hospital, Jalandhar, Punjab, India; n Arrhythmia Service, Cardiovascular Division, Department of Medicine, University of Minnesota, Minneapolis, MN, USA; o Dept. of Cardiology, National Heart & Lung Institute, Hammersmith Hospital Campus, Imperial College, London, United Kingdom

**Keywords:** Head-up tilt test, Syncope, Vasovagal syncope, Orthostatic hypotension, Psychogenic pseudosyncope

## Abstract

The Head-Up Tilt Test (HUTT) has been widely used for the past four decades as part of the overall assessment of the potential causes of collapse in patients with recurring transient loss of consciousness (TLOC) of unknown cause. The ability of a positive HUTT often to reproduce patient symptoms and illustrate to the patient that the physician is confident of the diagnosis have been major advances in clinical TLOC management. Tilt testing has been particularly important in understanding and diagnosing vasovagal syncope (VVS) and orthostatic hypotension.

Despite HUTT having great clinical utility, different HUTT protocols and drug provocations result in different test yields. Limited HUTT reproducibility has led some researchers to criticize HUTT utility. As in most medical tests, limitations are part of the test.

Herein, we provide a contemporary review of HUTT's utility in diagnosing and managing various TLOC disorders with intent to clarify its role in clinical practice.

**Table:** Abbreviations:

ACC	American College of Cardiology
AHA	American Heart Association
CRT	Cardiac Resynchronization Therapy
CSH	Carotid Sinus Hypersensitivity
CSM	Carotid Sinus Massage
ECG	Electrocardiogram
EEG	Electroencephalogram
ESC	European Society of Cardiology
HR	Heart Rate
HRS	Heart Rhythm Society
HUTT	Head-Up Tilt Test
ICD	Implantable Cardioverter Defibrillator
ICM	Implantable Cardiac monitor
ILR	Implantable loop recorder
ISO	Isoprenaline/Isoproterenol
IV	Intravenous
NTG	Nitroglycerine
OH	Orthostatic Hypotension
PCM	Physical – Maneuvers
POTS	Postural Orthostatic Tachycardia Syndrome
PPM	Permanent Pacemaker
PPS	Psychogenic Pseudo-syncope
SBP	Systolic Blood Pressure
T-LOC	Transient Loss of Consciousness
VASIS	Vasovagal Syncope International Study
VVS	Vasovagal Syncope

## 1 Introduction

Syncope is the commonest cause of transient loss of consciousness (T-LOC). By definition, syncope is attributable to transient self-terminating global cerebral hypoperfusion, and is estimated to account for around 1 % of all emergency visits and 3 % of hospital admissions.^1^^,^^2^ The lifetime cumulative incidence of syncope is ≥35 %, with a peak prevalence of the first episode between ages 10–35 years.^3^ Syncope is a common clinical problem, yet as much as 50 % of syncope remains undiagnosed.^4^

The most common cause of syncope across all age groups is vasovagal syncope (VVS) which is the most common form of the various neural reflex syncope disorders and is typically provoked by factors such as a hot environment, pain, and medical procedures. The immediate pre-VVS period may be associated with a prodrome consisting of nausea, pallor, diaphoresis. Syncope is brief (usually <2 min), and may be followed by drowsiness or fatigue.

While the medical history may in itself be diagnostic, often such symptoms are not present or not recalled (especially in older patients). Thus, even an experienced clinician may not be able to establish a VVS diagnosis with confidence. The Head-Up Tilt Test (HUTT) is helpful in such circumstances to evaluate the cause of presyncope and syncope when the diagnosis is in doubt. In other cases the ability of HUTT to reproduce symptoms may provide the patient confidence that the diagnosis has been established. HUTT may also be used for patient education in developing recognition of prodromes and therapeutic measures, notably physical counter-measures.^5^

HUTT utility is now well established for suspected VVS, but has also been applied for diagnosis of suspected orthostatic hypotension (OH), postural orthostatic tachycardia syndrome (POTS), psychogenic pseudosyncope, and neurogenic autonomic dysfunction (including cases believed to be of post-viral etiology).^6^

Despite HUTT having great clinical utility there are problems in performance and interpretation of the test that may adversely affect results. Different HUTT protocols and drug provocations result in different test yields, whether syncope or presyncope are induced and that the patient's volume status and autonomic conditions also influence test outcome. Limited HUTT reproducibility has led some researchers to criticize HUTT utility. Such negative aspects must be expected where the patient is told at the outset of the test that syncope may occur. Circumstances vary prior to spontaneous VVS which necessarily cannot be reproduced in the laboratory. Thus, limitations are part of the test as in most medical tests.

## 2 Purpose of this review

Although HUTT has been used for nearly 40 years in clinical practice. Its positive yield varies from 26 % to 87 %, with median sensitivity of 30 % and specificity of 90 %.^7^, ^8^, ^9^, ^10^, ^11^, ^12^ Various provocation protocols have emerged to increase its sensitivity, including sublingual nitrates and intravenous (IV) isoprenaline/isoproterenol (ISO). When patients with a positive HUTT are retested, approximately 50 % of tests are negative irrespective of treatment.^11^, ^12^, ^13^ The overall reproducibility of a negative response of 85 %–94 % is higher than that of an initial positive response of (31 %–92 %)^14^, ^15^, ^16^, ^17^, ^18^, ^19^, ^20^; there is however an estimated false-negative rate as high as 30 %; consequently the physician must consider test outcomes with care and relate findings to patient clinical presentation. False negative tests can be minimized by performing HUTT according to the criteria laid out in the ESC Guidelines^21^ which implies continuing the test until the patient has completely lost consciousness rather than stopping early. False positive tests occur in ∼13 % of normals who have never experienced syncope.^22^^,^^23^ In this instance, consideration should be given to the assertion that a positive HUTT is only showing a hypotensive tendency rather than making a definitive diagnosis.^24^ Medical tests seldom have high reproducibility, especially when they involve the autonomic nervous system. This was recognized as early as the European Society of Cardiology (ESC) guidelines of 2001.^25^

HUTT has received substantial criticism from some clinicians. It has even been suggested to abolish HUTT because of its inconsistent results.^26^ However, others^27^^,^^28^ have provided strong arguments favoring HUTT utility particularly when performed as recommended in ESC Guidelines^21^ and interpreted by experienced practitioners. In addition, more recently tilt has been shown clearly to reveal a hypotensive tendency, especially in the low blood pressure phenotype.^24^^,^^29^

It has been argued that variability of yield and reproducibility, variable protocols with poor adherence to them, lack of evidence for prognostic benefit or response to treatment, lack of randomized trials, time investment required and lack of reimbursement in some countries has resulted in HUTT falling out of grace in clinical practice. The manuscript by Kulkarni et al^26^ fails to give a balanced view of HUTT's position in contemporary clinical practice prompting opposition from syncope experts worldwide.^27^^,^^28^ Further, Kulkarni et al propose replacing HUTT by Active Stand which is essentially unstudied for this application and involves different hemodynamics.^28^ Further, availability of internal and external event recorders, and utilization of structured questionnaires to evaluate T-LOC have, probably inappropriately, led to HUTT falling out of grace in recent clinical practice.

The clinical history, always obligatory, but alone may not be adequate for establishing VVS diagnosis in all cases, and ‘competitive’ diagnostic methods such as questionnaires, and heart monitors have substantial drawbacks. In particular, available wearable monitoring systems are unable to detect blood pressure changes thereby unable to detect predominantly vasodepressor VVS events. Additionally, physicians often employ monitoring technologies suboptimally, and inadequate patient compliance may undermine diagnostic value. Consequently, HUTT remains a very useful tool, despite its apparent weaknesses, HUTT critiques have as yet not offered evidence favoring an alternative.^30^

## 3 Are tilt-induced syncope and spontaneous VVS comparable?

An important consideration is whether there is evidence to show that the response during tilt testing can be equated with a spontaneous VVS. The evidence, which assumes that underlying mechanisms of tilt-induced syncope and VVS are similar includes the following:

a) *Premonitory symptoms*: Both spontaneous vasovagal and tilt-induced syncope have similar premonitory symptoms and signs, including nausea, diaphoresis, and pallor, followed by the loss of postural tone.^3^^,^^4^ These symptoms may be recognized by the patient but, if not, they may be observed as physical signs by the operators of the test.^21^
b) *Hemodynamic changes:* Changes in blood pressure (BP) and heart rate (HR) during tilt-induced syncope closely parallel the symptoms during spontaneous VVS.^3^^,^^4^ There is an increased central sympathetic stimulation before syncope and an acute reduction in stroke volume associated with blood pressure fall^3^^,^^7^^,^^8^ which is known as vasodepression.
c) *Neuroendocrine changes*: There is a prominent increase in circulating plasma epinephrine during HUTT in VVS patients.^3^
d) A different catecholaminic pattern is seen during supine exercise^7^^,^^8^ which supports the theory that the abnormal release of epinephrine is part of the initiation of a vasovagal episode in addition to being a response to falling BP^3^^,^^9^^,^^10^^,^^31^

## 4 Responses to HUTT

The classification of responses and the methodology of HUTT have been well-described in the practical instructions released by the European Society of Cardiology (ESC).^32^ Table 1 shows the heart rate and blood pressure response during a positive HUTT.

**Table 1:** Response of HR and BP to HUTT.

Type of response	Change in BP on tilting	Change in HR on tilting
Normal or negative response^32^	No change in BP or a slight increase of <10 %	HR increased by < 10 %
Type 1 Mixed syncope^35^^,^^41^	BP decreases before HR fall	HR decreases at the time of syncope but does not reach <40 bpm for <10 s, with or without asystole <3 s
Type 2A Cardioinhibitory syncope without asystole^35^	BP decreases before HR fall	HR decreases <40 bpm for more than 10 s; without asystole >3 s
Type 2B Cardioinhibitory syncope with asystole^23^^,^^35^^,^^41^^,^^95^	Marked HR fall follows mild HR rise and BP fall after ∼8min.	Asystole >3 s
Type 3 Vasodepressor response^35^	SBP falls to <60 mm Hg	HR does not fall by >10 % of its peak value
	**Exception 1:** Chronotropic incompetence. No rise in HR during the tilt-table testing (<10 % from the pre-tilt rate)	
	**Exception 2:** Excessive rise in HR: both at the onset of the upright position and throughout its duration before syncope (>130 bpm)	
Initial/Immediate OH^21^^,^^33^	Decrease in BP > 40 mm Hg at standing with spontaneous and fast normalization so that hypotension and symptoms last <30 s. No HR fall.	
Classic OH^21^^,^^32^^,^^33^^,^^36^^,^^37^^,^^41^	Decrease in SBP >20 mm Hg and DBP >10 mm Hg during the first 3 min after standing. No HR fall.	
Delayed (progressive) OH^21^^,^^33^^,^^38^^,^^39^	Slow and progressive decline in SBP after 3rd minute of standing. No HR fall.	
POTS^21^^,^^33^^,^^40^^,^^41^	No BP fall	Increase in HR > 30 bpm or HR > 120 bpm after standing accompanied by symptoms and no BP fall
Psychogenic pseudo-syncope^21^^,^^33^^,^^41^^,^^51^, ^52^, ^53^, ^54^	No decreases in HR and BP; usually, both HR and BP increase several minutes before the event, reaching peak values during the event	

HR: heart rate; BP: blood pressure; HUTT: table-tilt test; bpm: beats per minute; POTS: postural orthostatic tachycardia syndrome; SBP: systolic blood pressure; DBP: diastolic blood pressure; OH: orthostatic hypotension.

The American College of Cardiology (ACC)/American Heart Association (AHA)/Heart Rhythm Society (HRS) defines HUTT as an orthostatic stress test to assess the susceptibility to a vasovagal response on postural change from supine to upright.^33^ This is correct but it lacks the breadth of approach given in the ESC Guidelines.^21^

1. Positive test for Vasovagal syncope

HUTT introduced clinically for diagnosis of unexplained syncope in 1986.^34^ In these early cases, the finding from induced syncope was clearly VVS. Subsequently, the outcomes of tilt were classified in the hope that therapy could be matched to each of these hemodynamic patterns. This was the Vasovagal syncope international study (VASIS) classification which was later modified to improve its clinical relevance.^35^

HUTT is considered positive if the patient has syncope with hypotension and/or bradycardia which is most convincing as a result when the patient states in reply to a non-leading question that what happened reproduced previous episodes.^21^^,^^28^^,^^32^^,^^33^ In many cases hypotension due primarily to venous capacitance changes is the cause of cerebral hypoperfusion. The latter cannot be adequately detected by ECG monitoring alone, whereas HUTT (during which both ECG and blood pressure are monitored) offers definitive evidence.

2. Orthostatic hypotension (OH)

A decrease in systolic blood pressure (SBP) ≥ 20 mm Hg and diastolic blood pressure (DBP) ≥ 10 mm Hg during the first 3 min after standing is diagnostic of OH.^36^^,^^37^ This is classic OH. There are two other types of OH; Immediate (iOH), also called initial, which occurs in the first 40s of being upright with the same numerical criteria. HUTT is not the best way to find this abnormality where Active Stand is better; secondly, there is Delayed OH (dOH) which begins only after 3 min of tilt which is hypotension with the same numerical criteria without the bradycardia of VVS.^38^^,^^39^

3. Postural orthostatic tachycardia syndrome (POTS)

POTS is characterized by palpitations, dizziness, and presyncope without substantial blood pressure fall related to orthostasis. HUTT or Active Stand test is necessary to confirm the diagnosis. HUTT in POTS is only necessary if the patient has had syncope (about 30 % of POTS patients have had syncope). In most cases, this will be found to be due to VVS. If no syncope is present, then HUTT is not necessary and 10 min of Active Stand will be all that is necessary.

The HUTT test is considered positive for POTS if the clinical symptoms are reproduced during HUTT with an increase in heart rate (HR) by ≥30 beats per minute (bpm), reaching ≥120 bpm, and is maintained during orthostatic stress.^40^ Other causes of tachycardia need to be excluded such as inflammatory diseases, anemia, pain etc. Many conditions will give this result. Careful physician assessment is needed, as in VVS.^6^

Skill of interpretation of tilt results is acquired by diligent observation of what is shown by continuous heart rate (ECG) and beat-to-beat blood pressure recording, together with symptoms and their relative timing of onset. Few texts have offered help with this. Some information is presented in the ESC practical guidelines^32^ and a more recent paper.^41^

4. Performance of HUTT as a diagnostic test.

The positive rate of HUTT is the highest (92 %) in patients with typical VVS, especially when syncope is associated with emotional triggers.^21^ The positivity rate has been reported to be 73 %–78 % in patients with situational syncope (micturition, cough and many others) tested with nitroglycerin (NTG) protocol.^21^^,^^42^ When HUTT is performed with NTG provocation, a positive response is seen in 47 % of patients with cardiac syncope, 33 %–35 % with unexplained syncope, and 10 % with no syncope.^21^^,^^24^ The tilt test is often unhelpful in patients where a diagnosis is most needed.^24^ These cases are frequently those where structural heart disease exists and syncope is unexplained. A finding of VVS depends much on the patient's comparison of the HUTT result with previous episodes. It must always be recalled that HUTT is merely demonstrating a hypotensive susceptibility which may be interpreted as VVS.^24^ Sadly, there is no Gold standard test in syncope. Hypotensive susceptibility may be present even in patients with cardiac syncope and may help to determine therapeutic interventions.^24^

## 5 Indications and contraindications for HUTT

### 5.1 Indications for the HUTT

• A single syncopal episode in a high-risk setting that might cause physical injury
  o OR multiple episodes in the absence of structural cardiac disease
  o OR patients with structural cardiac disease when cardiac syncope is considered unlikely
• Recurrent unexplained falls
• Susceptibility to reflex syncope
• Discrimination between OH and reflex syncope
• Carotid sinus syndrome or carotid sinus hypersensitivity
• Differentiate syncope from epilepsy especially when abnormal movements occur during loss of consciousness
• Frequent syncope (many times per day or per week) suggesting psychogenic pseudosyncope.

### 5.2 Contraindications for HUTT

The contraindications for performing the tilt test include significant carotid artery stenosis, significant coronary artery stenosis, hypertrophic cardiomyopathy with outflow tract obstruction, severe anemia, hemodynamically significant aortic stenosis, known origin of the syncopal event, and unstable conditions like acute coronary syndromes. Pregnancy should be considered to be a relative contraindication to HUTT, bearing in mind that pregnancy militates against VVS due to its associated increase in blood volume. However, in later pregnancy the uterus may temporarily obstruct venous return from legs and pelvis. Tests can be safely performed if safety is carefully observed with duration of asystole minimized by fast tilt down in <15s. A positive test with symptom reproduction may prevent the undertaking of an electrophysiological study.

## 6 Clinical utility of HUTT

HUTT is mainly used to establish a diagnosis of reflex vasovagal (VVS) syncope in patients in whom this diagnosis is suspected but not confirmed by initial evaluation^1^^,^^7^, ^8^, ^9^, ^10^^,^^21^. HUTT can be considered for patients with suspected recurrent VVS, OH, POTS, psychogenic pseudosyncope (PPS)*,* carotid sinus syndrome (CSS) but not Carotid sinus hypersensitivity (CSH) which is positive carotid sinus massage without a history of syncope,^21^ and may be used in primary & secondary autonomic disorders, where Active Stand is a viable alternative.^28^^,^^32^

### 6.1 In patients with true vasovagal syncope or without a history of syncope

Tilt testing has an acceptable sensitivity^43^ and specificity^23^^,^^43^^,^^44^ in patients with true VVS or those without a history of syncope. A positive tilt test reveals susceptibility to orthostatic stress.^24^ Positive HUTT in different clinical conditions and with various protocols are summarized in Table 2. These studies used the Westminster protocol - passive tilt,^12^ the Italian protocol including NTG,^44^ and the clomipramine protocol.^43^ Most recently, the short Italian Protocol^45^ has matched results with the longer Italian protocol and saved considerable laboratory time. Studies using other tilt protocols, e.g., isoproterenol challenge, were not included.

**Table 2:** Tilt-table positivity.

Clinical condition	Drugs used	Positivity (%)
Subjects without syncope	Passive tilt^32^ NTG^21^^,^^33^^,^^69^, ^70^, ^71^, ^72^, ^73^, ^74^, ^75^, ^76^ Clomipramine^43^	8–13 %
Unexplained syncope	NTG^21^^,^^33^^,^^44^^,^^45^ Clomipramine^43^	30–36 %
Likely tachyarrhythmic syncope	Passive^21^	45 %
Cardiac syncope	NTG^21^^,^^24^	47 %
Likely a reflex, atypical	NTG^21^^,^^33^^,^^44^^,^^45^	51–56 %
Typical VVS, Miscellaneous	NTG^21^^,^^33^^,^^44^^,^^45^ Clomipramine^43^	65–73 %
Typical VVS, situational trigger	NTG^21^^,^^33^^,^^42^	78 %
Typical VVS, emotional trigger	Clomipramine^43^	92 %

NTG: nitroglycerin; VVS: vasovagal syncope.

When used with a structured history and physical exam, HUTT sensitivity approaches 78–92 % with a specificity of 92 % by current recommended protocols.^21^^,^^28^^,^^33^ Adding pharmacological provocation using sublingual NTG, ISO, or clomipramine increases the positive response and improves the diagnostic value of the test^43^^,^^46^, ^47^, ^48^ The endpoint of tilt testing is the reproduction of symptoms with reflex hypotension/bradycardia. In patients with OH, POTS, or PPS, the endpoint is the reproduction of symptoms with the characteristic heart rate or blood pressure response as described.^21^^,^^33^

### 6.2 In the diagnosis of orthostatic hypotension

Orthostatic hypotension is classified as immediate/initial, classic or delayed. Classic OH occurs within 3 min of standing or during HUTT. Delayed OH is hypotension after 3 min, but usually after prolonged standing.^38^^,^^39^ In a 10-year follow-up study by Gibbons et al.^38^ in 165 patients, 54 % of cases diagnosed as delayed OH by tilt table testing progressed to classic OH with time. The 10-year mortality rate in patients with delayed OH is 29 % compared to 9 % in controls.^39^ Tilt in OH has recently been reviewed with helpful recommendations.^49^

In classic and delayed OH, it is necessary to see a broader picture by performance of the Active Stand test (HUTT may not always be necessary) and other autonomic tests including Valsalva maneuver, respiratory tests and ambulatory blood pressure monitoring.^32^

### 6.3 In patients with carotid syndrome - CSS

Performing the carotid sinus massage (CSM) during passive tilt increases the diagnostic yield of CSS/CSH. The test is positive if the patient develops syncope with ventricular asystole ≥3 s or falls in SBP ≥50 mmHg. CSS is revealed by this result if the patient has had previous syncope. If there has been no previous syncope the diagnosis is CSH. In a retrospective study by Puggioni E et al.^50^ in 1719 patients, carotid sinus syndrome was diagnosed in 226 patients when CSM was performed in the supine position, and further 217 patients were diagnosed after CSM was repeated with upright posture on the tilt table. Continuous BP monitoring during HUTT reveals the vasodepressor component of the syncope when there is a simultaneous fall in HR, BP, or a mixed response. The 'method of symptoms' employing use of IV Atropine, which eliminates the cardioinhibitory aspect and thereby helps to define the type of response.^21^^,^^32^

### 6.4 Psychogenic pseudosyncope (PPS)

HUTT helps to diagnose PPS.^51^, ^52^, ^53^ Compared to VVS, PPS patients are usually female, there may be a history of physical and/or sexual abuse, eye closure and lack of pallor and diaphoresis and increased HR and BP are highly specific for PPS during the event, long periods of apparent transient loss of consciousness (T-LOC), absence of prodromes. Notably, the patient is unresponsive without hypotension or bradycardia and, if included, a normal EEG. EEG recording during HUTT establishes the diagnosis as the EEG is normal despite the patient being unresponsive.

### 6.5 Role of HUTT in diagnosing a true seizure and separating seizure from vasoval syncope with abnormal movements

VVS can present with jerky (tonic-clonic) movements, they are essentially irregular in nature. This is sometimes termed Convulsive syncope which is an erroneous and confusing term which we prefer not to be used. Prolonged cerebral hypoxia and convulsions during VVS can lead to jerky irregular movements often misdiagnosed as a convulsion or seizure. HUTT helps to differentiate between VVS and epilepsy.^54^, ^55^, ^56^, ^57^, ^58^, ^59^, ^60^ During tilt, reproducible spontaneous prodrome symptoms and jerky (abnormal) movements associated with a typical vasovagal reflex support the diagnosis of VVS and deny Epilepsy. Such movements differ from seizures in their quality and number (<10 in VVS vs > 20 in epilepsy).^55^

Some of these attacks may be ictal seizures which are combinations of temporal lobe epilepsy and triggering of VVS. HUTT may be helpful in differentiating VVS with convulsive movements from actual epileptic seizures.^55^, ^56^, ^57^ Approximately half of the cases of drug-refractory or questionable seizures may be undiagnosed VVS.^58^ A focused history and examination along with an electroencephalogram (EEG) may point to the correct diagnosis. Both Psychogenic non-epileptic seizures and pseudo syncope have normal EEGs.^61^ Abnormal discharges are recorded in epileptic convulsions, whereas, in syncope, an EEG shows diffuse slowing followed by a flat line.^55^^,^^58^

Normal BP, HR, and EEG when the patient appears unconscious (unresponsive) rule out true syncope and most forms of epilepsy.^56^^,^^58^ In a study of 21 patients by Zaidi and colleagues, 81 % had typical symptoms during HUTT without significant electroencephalographic abnormalities or hemodynamic changes.^61^

## 7 Protocols for HUTT

HUTT was first introduced to clinical practice in 1986 by Kenny et al.^34^ The patients were fasted overnight. They were subjected to a 40° head-up tilt initially for 120 min, later 60 min and their heart rates and SBP were continuously monitored, pharmaceutical provocation was not used. These previously symptomatic patients showed a fall in SBP followed by HR leading to syncope during the test. There was reproduction of earlier symptoms.

### 7.1 Westminster protocol

Fitzpatrick et al described the Westminster protocol in 1989.^62^ Tests were performed after overnight fasting and withholding medications for three half-lives. In this protocol, the tilt table was elevated at 4°/second to 60° angle, which was maintained for 60 min. The duration of tilt was intended to be 60 min but was discontinued in the event of syncope. The heart rate and BP recording were recorded at baseline and 5-min intervals; later in the series beat-to-beat BP was included. Prolonged tilt duration increases specificity to 90 % in patients with recurrent unexplained syncope. Fitzpatrick^12^^,^^62^ recommended reducing the duration of tilt to a minimum of 45 min which represented 2-standard deviations from the mean time to syncope of 23s. The test did not include provocative agents, and the reported sensitivity was 75 %. Baron-Esquivias et al reported a 41 % positive rate in young patients <20 years and 19 % in patients >60 years.^63^

### 7.2 Provocative protocols

Subsequently, different provocative protocols were described to improve the test's sensitivity and specificity and reduce test duration. The commonly used provocative protocols are described below:

#### 7.2.1 Isoprenaline infusion protocols

Almquist et al, based on prior work by Waxman and colleagues^64^^,^^65^ described the use of isoproterenol (ISO) infusion during HUTT. In this protocol, the patient is tilted to 80° for 10 min at baseline. If the test is negative, isoprenaline (ISO) infusion is started at 1 μg/min and increased stepwise to 5 μg/min. The ISO protocol has a sensitivity of 87 % and a specificity of 85 %.^66^ Carlioz et al reported the use of low-dose ISO during the HUTT. Low-dose ISO infusion (<3 μg/min) improved the sensitivity with sufficient specificity.^67^ Shen et al described a shorter duration single-stage protocol using a low-dose ISO infusion. In this protocol, after 5 min in the supine position, the patient is tilted to 70° angle for 10 min on low-dose ISO. This protocol has a specificity of 83 % and was recommended as an alternative.^68^

#### 7.2.2 Nitroglycerine (NTG) protocols

Raviele et al studied the use of NTG infusion in 40 patients during HUTT.^69^ The protocol consisted of 5 successive stages of 5 min in the supine position plus 10 min of 80^o^head-up tilt with progressively increasing rates of NTG infusion. This infusion was increased in increments of 0.86 μg/kg/hr at every stage. They concluded that the NTG protocol was equally specific (92 %) but more sensitive (53 %) than the ISO test. Raviele et al later described HUTT using sublingual NTG.^10^ Patients were tilted at 60° for 45 + 20 min. The initial 45 min were without medications, and the next 20 min were followed by 300 μg of sublingual NTG. The authors concluded that the addition of NTG doubled the sensitivity of the upright tilt from 25 % to 51 % and had a high specificity of 94 %. The Newcastle, UK group^70^ suggested front-loading tilt with early drug administration but this has not been widely adopted.

Subsequently, many investigators turned to use of NTG.^71^ Bartoletti et al described the simplified Italian protocol in 2000.^44^ In this protocol, after a stabilization phase of 5 min in the supine position, the subject is tilted to a 60° angle for 20 min in the passive tilt phase. If the test is negative in the passive phase, it is followed by a provocative phase of 15 min after 400 μg of sublingual NTG spray.^44^ Venous access is deemed unnecessary and is avoided so as not to influence the test response. The use of NTG spray reduces the time to syncope compared to sublingual NTG tablets. The Italian protocol is simple and fast and mentions the pre-tilt table phase to be shortened to 5 min in case of no venous access. The Italian protocol has a sensitivity of 62 % and a specificity of 92 %. In 2023, Russo et al^45^ presented the short Italian Protocol with 5 min supine pretest, 10 min passive phase and 10 min NTG phase. Comparing the short with the original Italian protocols in two cohorts of >250 patients showed no significant difference in outcome.

Numerous studies have compared the tilt table protocols using ISO and NTG protocols.^72^, ^73^, ^74^, ^75^, ^76^ (Table 3).

**Table 3:** Studies comparing the tilt-table protocols using ISO and NTG protocols.

	Sublingual NTG	Sublingual NTG	Sublingual NTG	ISO	ISO	ISO
	**Positive Response**	**Negative response**	**Drug intolerance**	**Positive Response**	**Negative response**	**Drug intolerance**
**Raviele et al**^43^	49 %	51 %	0	41	59	6
**Jimenez-Chol et al**^74^	75.5 %		22 %	77.6 %		38 %
**Prabhu et al**^75^	48.4 %		0.12 %	35.9 %		1.6 %

	**Sensitivity**	**specificity**		**Sensitivity**	**Specificity**	

**Graham et al**^73^	68 %	71 %	0 %	52 %	64 %	80 %

NTG: nitroglycerin; ISO: isoproterenol.

A call for standardization of HUTT is appropriate. Isoproterenol must be given intravenously which carries risks of pain at insertion disturbing the autonomic milieu and the well-documented adverse effects on cardiac arrhytmias. Its recent massive increase in price implies that is now outmoded. Nitrogylcerine is cheap, easily administered and without serious side effects. HUTT can now be used in the short Italian protocol^45^ which enhances productivity without affecting yield. It is with this methodology that HUTT should be standardized.

The most recent development in HUTT methodology is strictly not in HUTT itself but in associated tests attempting to adopt a broader view of the presenting patients. It includes with HUTT CSM as previously mentioned, Active stand, also mentioned, and some cardiovascular autonomic tests, Valsalva maneuver, deep breathing and ambulatory blood pressure monitoring (ABPM) which offer definitive etiology of syncope in >80 % of patients.^77^, ^78^, ^79^ It is noteworthy that ABPM is the first test to show that autonomic abnormalities exist even in VVS patients.^77^

#### 7.2.3 Other agents used for provocation

Other agents like IV edrophonium and clomipramine have been used for provocative testing. However, they are not widely accepted due to unfamiliarity with these agents.^43^^,^^80^^,^^81^ Clomipramine use is recommended for HUTT in patients with emotional triggers.^77^ In a semi-cross-over randomized trial to compare NTG and adenosine (AD) as provocative agents, no statistically significant difference was noted in positive results; however, the time to positive response was significantly shorter for AD than NTG. The authors concluded that the diagnostic yield of NTG and AD in HUTT is comparable, while AD acts 4 times faster than NTG in evoking a vasovagal response.^82^ HUTT with NTG provocation has a higher positive response, lower incidence of unwanted arrhythmias, and better tolerability than ISO. VASIS^35^ type IIb and type III response was more with NTG than ISO(75).

#### 7.2.4 ACC*/AHA/HRS and ESC recommendations*

All major North American societies, ACC/AHA/HRS in 2017, ESC in 2018 recommend HUTT as one of the tests for the work-up of syncope. Though both guidelines reference the protocols, both include a short 20-min unmedicated phase, a tilt table angle of 60°-80° and provocative testing with either low-dose ISO or sublingual NTG (Table 4).

**Table 4:** Comparative recommendations from the ESC and ACC/AHA/HRS guidelines.

Parameter	ESC guidelines^21^^,^^32^	ACC/AHA/HRS guidelines^36^
	20-min unmedicated phase	
Tilt table angle	60–70°	70°
Duration	20–45 min	30–40 min
Provocative agents	Low dose ISO until average HR increases by about 20–25 % over baseline (usually≤3 μg/min)	Low dose ISO
	Sublingual NTG	Sublingual NTG

HR: heart rate; NTG: nitroglycerin; ISO: isoproterenol; ESC: European Society of cardiology; ACC: American College of Cardiology; AHA: American Heart Association.

The ESC guidelines on HUTT recommend^21^

● Patients fast for 2–4 h before the test
● A supine pre-tilt phase of 5 min if there is no venous access and 20 min if a venous cannula is inserted
● The tilt angle of 60°-70^o^
● Passive phase tilt of 20–45 min
● The provocative phase of 15–20 min
● NTG challenge: 300–400 μg sublingual
● ISO challenge: incremental infusion from 1 μg/min to 3 μg/min to increase average heart rate by about 2 %–25 % over baseline
● The test continued till completion of the protocol or till loss of consciousness

The ACC/AHA/HRS guidelines on syncope recommend^33^

● Tilt table angle of 70^o^
● Duration of 30–40 min
● Provocation agents recommended: low dose of ISO infusion or sublingual nitrates

#### 7.2.5 Our recommendations for the tilt-table protocols

Though there is no standardized protocol for HUTT, drug-free protocols have the highest specificity, while provocative protocols have higher sensitivity. It is strongly advisable to use beat-to-beat non-invasive BP monitoring for HUTT. The non-invasive measurements are done by finger cuff using the volume-clamp technique (Finapres Medical systems, Enschede, the Netherlands). Changes in intra-arterial pressure are closely followed with 89 % of beat-to-beat changes in systolic pressure, and 95 % of beat-to-beat changes in diastolic pressure followed to within ±2 mmHg. Thus, a non-invasive beat-to-beat BP measurement is accurate in these circumstances.^83^ Provocative testing with both NTG and ISO are popular all over the world. The Short Italian protocol has already gained traction in Europe.^45^ We recommend carrying out provocative testing either with NTG during HUTT. An IV line for HUTT is not required and may even influence the subsequent results of the test by causing pain. If it is felt that an IV line is a necessity, it should be placed at least 20 min before commencement of the HUTT which will avoid the bulk of the following autonomic disturbance. Testing with sublingual NTG reduces the testing time and avoids IV access, is easy to carry out, and is better tolerated^44^^,^^45^^,^^75^^,^^76^ and thus may be more suited for our clinical and hospital settings. Also, NTG is substantially less expensive than ISO in most countries.

#### 7.2.6 Laboratory setup

The laboratory setup consists of a automated tilt-table bed which can tilt with the single press of a button. The table needs to have retention straps of Velcro to prevent the patient from following in case of syncope and loss of postural tone. The Tilt table needs to tilt up to angles of 60°-80°. The table must be able to be tilted down to supine within 15s to avoid prolongation of syncope.^84^ ECG should be continuously monitored and recorded using a 3-lead recording system. Its preferable to use a beat-to-beat BP monitoring system. Video recording of the same is preferable but optional. Detailed reviews of the laboratory setup have been described elsewhere.^41^^,^^85^

## 8 Probability of syncope recurrence after positive HUTT

Tilt testing may give insights into future recurrences and the correlation of the test to the clinical occurrence. Singhania et al found that time to tilt positive response of <19.5 min predicted a high likelihood of future syncope.^86^ A recent sub-analysis from ISSUE-3 found that asystole during HUTT predicted a recurrence rate of 5 % in patients with negative tilt test results compared to 55 % in those with positive tilt test results.^87^ An unexpected finding of the ISSUE-3 study was that patients who were tilt-positive benefitted little from pacing, while those who were tilt-negative with the same ILR findings received benefit in terms of a significant reduction in syncope recurrence after pacing.^88^ This finding is thought to represent the role of vasodepression.^24^

Various investigators have studied if the probability of syncope increases after a positive HUTT.^89^ Sheldon et al followed 101 patients with a positive HUTT to identify patients at high risk for recurrent syncope. The number of pre-test syncopal spells and their durations were the main predictive factors with a high chance of syncope recurrence after the tilt test. Patients who developed syncope and presyncope during the tilt test have a probability of remaining free of syncope of 0.45 and 0.72, respectively, over 2 years.^89^ A study by Yilmaz et al also found a higher recurrence of syncope in patients who had syncope in the first 20 min of the test.^90^ Yilmaz et al noted that the number of syncope episodes reduced after the HUTT but this was independent of the test results.^90^ However, Grimm et al did not find an increased incidence of syncope in patients with positive tilt tests.^91^In another study, the results of the HUTT did not predict the future occurrence of syncope.^92^ In the ISSUE-2 trial, positive HUTT results did not predict future syncope episodes by univariate analysis.^93^

A recent observation in HUTT methodology is, that in previously syncopal patients, it is necessary to prolong the upright posture until consciousness is lost.^94^ This gives the patient the best chance of recognizing a previous event and shows better occurrence of asystole which will help to guide therapy in older patients when permanent pacing is being considered, as in the SPAIN^95^ and BioSync^96^ trials. Both trials selected their patients on the basis of documented cardioinhibition and found clear benefit of pacing versus no pacing. These trials have caused the ESC, in their 2021 pacing guidelines, to class permanent dual pacing in older (>40 years) patients refractory to conventional measures with asystole on HUTT as a Class I A indication.^97^

## 9 Safety and cost-effectiveness of HUTT

HUTT is a safe test when performed as per recommendations and after excluding cardiac causes of syncope when necessary. Large studies have shown that HUTT is not only safe^21^ but also has rare complications.^21^ There are rare reports of ventricular fibrillation during HUTT with ISO and NTG provocation in patients with undetected coronary artery disease.^98^^,^^99^ ISO may precipitate coronary vasospasm.^100^ HUTT should be avoided in patients with reversible ischemia. Prolonged asystole or hypotension may occur during HUTT but is easily reversed by the supine or Trendelenburg position,.^21^^,^^101^ Resucitation equipment should be available but will hardly ever be required. Other rare adverse events are ventricular arrhythmias, transient atrial fibrillation, in all protocols.^21^ Minor side effects like headaches and palpitations are commonly due to drugs, and spontaneously resolve.^21^ HUTT has been shown to be safe in an older population,^102^ long-term outcomes are benign^103^ even when relatively aggressive protocols are used.^103^

HUTT is a cost-effective method for supporting a clinical suspicion of reflex syncope, yielding a firm diagnosis, when there is no cardiovascular disease. Tilt testing is indicated if other non-invasive modalities cannot establish the etiology. According to Krahn et al, “tilt table testing was the most cost-effective strategy at $2,901 per diagnosis, followed by the implantable loop recorder ($6,158 per diagnosis), external loop recorders and EP testing”.^104^ In patients with, T-LOC with seizure-like phenomenon and normal EEGs, it is also cost-effective to do a HUTT.

## 10 Why not an implantable cardiac monitor (ICM) instead of HUTT?

This matter has been discussed and practiced recently. It can be addressed simply; ICMs (ILRs) are unable to record blood pressure, thus, there is no indication of vasodepression which is a integral part of VVS. This feature will be added some time in the future. Secondly, ICMs are very expensive and cannot be compared in cost-effectiveness with HUTT although opinions differ on this matter.^105^

Nevertheless, there is much that can be learnt about VVS from ICMs. In the early 1990s, Brignole and co-workers showed that bradycardia (cardioinhibition) is more common in spontaneous attacks of VVS seen on an ICM than on tilt.^22^ These findings were later qualified by Deharo and colleagues.^106^ The advent of blood pressure recording will change our perception of this choice but until this becomes available HUTT is favored over ICM.

## 11 Future directions

The role of HUTT has fallen out of grace in many cardiology departments due to its cumbersome technique, limited reproducibility, and growing confidence with implantable loop recorders (ILR/ICM). However, with less time-consuming tests^45^ and increasing evidence from both the SPAIN^95^ and BioSync^96^ trials where tilt showed cardioinhibition and was a major selection criterion for pacing with success in patients of >40 years. Further, in the current practice of cardioneuroablation documented cardioinhibitory VVS is considered to be absolutely necessary^107^^,^^108^. This therapy has so far largely been confined to younger individuals. HUTT, therefore, will continue to remain a critical diagnostic and risk stratifying test.

## 12 Conclusion

HUTT continues to be widely used and retains significant utility in investigating unexplained TLOC. It is a low-risk, non-invasive test that, despite limitations, when used in the appropriate clinical context and following established guidelines, remains valuable in managing patients with unexplained syncope. To maximize this test's value in clinical practice, physicians must be aware of its limitations and correlate clinical information and other testing data with the tilt table test results. Further, as with any test in medicine, the results must be interpreted by an experienced practitioner in the context of the patient's clinical presentation.

## Funding sources

None.

## Declaration of competing interest

The authors declare that they have no known competing financial interests or personal relationships that could have appeared to influence the work reported in this paper.
